# *Lactococcus cremoris* YRC3780 improves subjective stress response in the Uchida-Kraepelin test: a randomized, double-blind, placebo-controlled study

**DOI:** 10.1038/s41598-025-07783-z

**Published:** 2025-07-02

**Authors:** Ikumi Fujioka, Kenji Uchida

**Affiliations:** R&D Center, Yotsuba Milk Products Co., Ltd, 465-1 Wattsu, Kitahiroshima, Hokkaido 061-1264 Japan

**Keywords:** Microbiology, Health care

## Abstract

*Lactococcus cremoris* YRC3780, isolated from kefir, likely improves the hypothalamic–pituitary–adrenal axis response to acute psychological stress by acting on intestinal immune cells and promoting cytokine production. Here we examined the effectiveness of YRC3780 on the stress response in healthy Japanese adults. We investigated the effects of daily YRC3780 intake on the stress response in a randomized, placebo-controlled, double-blind, parallel-group study of 107 healthy Japanese adults (54 in the YRC3780 group and 53 in the placebo group) who had an initial positive Uchida-Kraepelin (U-K) test stress response. After 8 weeks, the POMS2 Total Mood Disturbance (TMD) score after the U-K test and the change in score from baseline score were significantly better in the YRC3780 group than in the placebo. The YRC3780 group showed significant improvements in the POMS2 TMD and in five POMS2 subscores difference before and after the U-K test at 8 weeks. Daily YRC3780 intake significantly improved the stress response in healthy Japanese adults who had an initial stress response in the U-K test.

## Introduction

Today’s society is considered stressful, and 82.2% of Japanese workers feel that there are things related to their current work or work life that cause them strong anxiety or stress (Reiwa 4 Occupational Safety and Health Survey^1^). The stress response is a natural adaptive response to external changes, but whether or not it is strong varies among individuals. If excessive stress persists, it can affect mental and physical health; because stress is difficult to eliminate, it is important for us to know how to deal with it and to thus reduce the risk of disease. As a coping strategy, it is important for us to understand the stresses we are subjected to, to relieve or reduce that stress in moderation, and to accelerate our recovery from the stress response.

Reports addressing the benefits of probiotic intake on the stress response are increasing. These benefits are assumed to occur through gut–brain interaction; signaling molecules from the gut to the brain include short-chain fatty acids (e.g., butyric acid) produced by intestinal bacteria, serotonin released from chromaffin cells in the small intestine, and cytokines produced by immune cells^2–4^. In particular, *Lactococcus cremoris* YRC3780 (hereafter, “YRC3780”), which is isolated from kefir, significantly increases IL-2 production by, and natural killer cell activity in, the spleens of colon-cancer-bearing mice^5^. In addition, YRC3780 consumption has alleviated the symptoms of birch pollinosis^6^ and perennial allergic rhinitis^7^ in healthy human adults, with decreased TARC (thymus and activation-regulated chemokine) production and a trend toward enhanced blood interferon-gamma levels^7^. Therefore, YRC3780 may act on immune cells in the intestinal tract to enhance cytokine production, and these cytokines in turn may improve the stress response.

A study of men in their 20 s confirmed that ingestion of YRC3780 for 8 weeks changed diurnal fluctuations in salivary cortisol, improved subjective sleep quality, and improved mental health. In addition, in a Trier Social Stress Test (TSST) conducted at 8 weeks as part of the study, salivary cortisol remained low in the YRC3780 group, and cortisol levels 40 min after the start of the test were significantly lower than those in the placebo group^8^. The stress response during the TSST (elevated cortisol concentration = hypothalamic–pituitary–adrenal stress response) is affected by age, sex, and (in women) the menstrual cycle^9–12^.

A study of women reported that the stress response was observed only in the follicular phase and that the response was weaker in the luteal phase^13^. Moreover, in support of this finding, a trial that examined the difference in within-test reactivity between morning and evening TSSTs confirmed that reactivity in women did not differ from that in men when the TSST was administered in the follicular phase^12^. In the earlier TSST study^8^, it was considered difficult to adjust the timing of the TSST to each subject’s menstrual cycle, so the trial was conducted only in men and only in a group in their 20s.

In this study, we expanded the range of age and gender of the subjects, used the U-K test, which is easy to accomodate to in a wide range of age groups, rather than TSST. As an objective measure, we used salivary cortisol, which was conducted in previous studies. In addition, we also used the Profile of Mood States (POMS) as a subjective measure.

## Results

### Participants

**Fig. 1:**
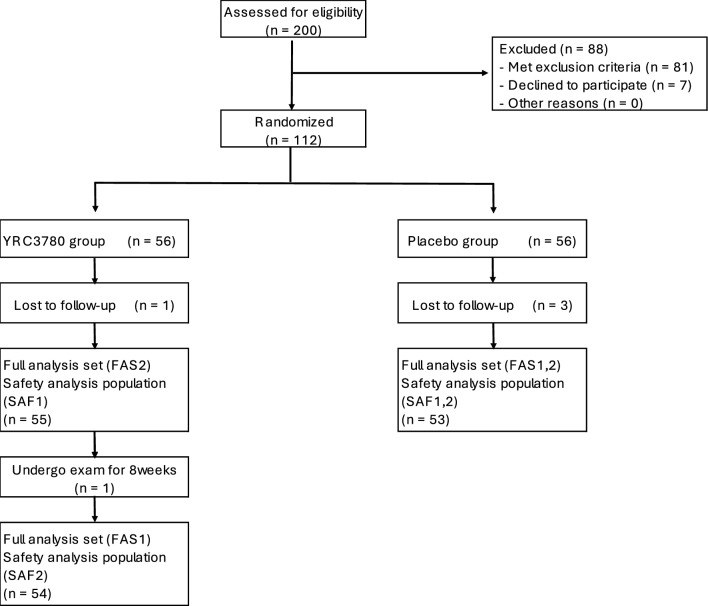
Flow chart of participant recruitment and participation.

The 112 participants who met the eligibility criteria were enrolled in the study and assigned to receive either YRC3780 or the placebo (*n* = 56 per group). All participants received the allocated intervention, but 4 participants have not received any intervention after allocation. Therefore 4 participants were excluded from the analysis, for a final count of 108 participants (55 in the YRC3780 group and 53 in the placebo group) constituted the full analysis set (FAS2) and safety analysis set (SAF1). In addition, 1 participants was not undergo the post 8 weeks and therefore was excluded from the analysis for FAS2, for 107 participants (54 in the YRC3780 group and 53 in the placebo group) constituted the full analysis set (FAS1) and safety analysis set (SAF2). Figure 1 shows the participant flow throughout the study; Table 1 summarizes the backgrounds of the participants and their test meal consumption rates.

**Table 1:** Participant information.

	Placebo (n = 53)	YRC3780 (n = 54)
Sex, no. males/no. females	28/25	28/26
Age, years	44.2 ± 12.0	44.1 ± 13.6
Height, cm	165.5 ± 8.8	164.8 ± 8.1
Weight, kg	64.4 ± 13.4	61.3 ± 14.1
Body mass index	23.5 ± 4.7	22.4 ± 4.0
Systolic blood pressure, mmHg	115.6 ± 18.6	117.4 ± 17.7
Diastolic blood pressure, mmHg	74.0 ± 12.1	76.0 ± 11.7
TMD score before U-K test at baseline	56.9 ± 9.2	56.7 ± 11.2
TMD score after U-K test at baseline	60.9 ± 10.3	60.3 ± 11.6
TMD score difference before and after U-K test at baseline	4.0 ± 4.4	3.6 ± 3.4
Test-meal consumption, %	99.9 ± 4.0	100.5 ± 3.4

Data are given as means ± SD.

TMD, the POMS2 total mood disturbance score.

### POMS2

The primary efficacy outcome—the POMS2 TMD score after the U-K test at 8 weeks—and the change in score from baseline were significantly lower in the YRC3780 group than in the placebo group (Table 2). This was also the case for the POMS2 subscore (AH, CB, DD, FI, TA) difference before and after the U-K test at 8 weeks and the change in score from the baseline were significantly lower in the YRC3780 group than in the placebo group (Table 3). Moreover, POMS 2 subscore (DD, TA), percentage change from baseline were significantly lower in the YRC3780 group than in the placebo group (Table 3). POMS TMD and subscore(AH, CB, DD, FI, TA) during intake period were shown in Table 4.

**Table 2:** POMS2 TMD (total mood disturbance) scores and subscores after the Uchida-Kraepelin (U-K) test at 8 weeks and its change from baseline.

	Placebo		YRC3780	
	Baseline	Week 8	Baseline	Week 8
TMD score after U-K test	60.9 ± 10.3	53.2 ± 10.8	60.3 ± 11.6	50.0 ± 9.7 *
Change in score from baseline (points)	—	–7.7 ± 6.0	—	–10.4 ± 9.0 *
Percentage change from baseline (%)	—	–12.6 ± 9.7	—	–16.2 ± 12.5
AH score difference after U-K test	57.0 ± 11.3	50.5 ± 11.3	54.9 ± 11.9	47.0 ± 9.3
Change in score from baseline (points)	—	–6.5 ± 10.8	—	–7.9 ± 11.0
Percentage change from baseline (%)	—	–10.0 ± 18.8	—	–12.5 ± 16.5
CB score difference after U-K test	61.2 ± 12.4	53.8 ± 12.1	62.2 ± 12.6	51.9 ± 10.7
Change in score from baseline (points)	—	–7.4 ± 5.8	—	–10.3 ± 10.4
Percentage change from baseline (%)	—	–11.9 ± 8.9	—	–15.3 ± 14.1
DD score difference after U-K test	58.9 ± 11.7	51.8 ± 10.4	57.6 ± 12.2	48.7 ± 9.3
Change in score from baseline (points)	—	–7.1 ± 6.9	—	–8.9 ± 9.5
Percentage change from baseline (%)	—	–11.2 ± 11.1	—	–14.0 ± 13.9
FI score difference after U-K test	62.1 ± 9.3	53.7 ± 11.0	61.3 ± 10.9	51.1 ± 11.4
Change in score from baseline (points)	—	–8.4 ± 7.6	—	–10.2 ± 10.1
Percentage change from baseline (%)	—	–13.5 ± 12.4	—	–15.9 ± 14.6
TA score difference after U-K test	59.7 ± 10.5	51.7 ± 10.7	59.8 ± 11.5	49.9 ± 10.5
Change in score from baseline (points)	—	–8.1 ± 7.2	—	–9.9 ± 9.2
Percentage change from baseline (%)	—	–13.1 ± 11.7	—	–15.8 ± 13.8
VA score difference after U-K test	45.2 ± 9.1	45.7 ± 9.6	45.0 ± 9.0	47.3 ± 9.2
Change in score from baseline (points)	—	0.5 ± 7.2	—	2.2 ± 7.9
Percentage change from baseline (%)	—	2.1 ± 15.7	—	6.6 ± 16.9
F score difference after U-K test	46.8 ± 10.8	45.2 ± 11.3	43.2 ± 11.2	44.2 ± 10.4
Change in score from baseline (points)	—	–1.6 ± 8.6	—	1.1 ± 9.3
Percentage change from baseline (%)	—	–2.0 ± 18.0	—	5.5 ± 21.8

Data are shown as means ± SD (Placebo, n = 53, YRC3780, n = 54).

Significant differences (*p < 0.05, ANCOVA with each initial value as a covariate) between the placebo and YRC3780 groups are indicated.

TA, Tension–Anxiety, DD, Depression–Dejection, AH, Anger–Hostility, CB, Confusion–Bewilderment, AH, Anger–Hostility, VA, Vigor–Activity FI, Fatigue–Inertia, F, Friendliness.

**Table 3:** POMS2 TMD scores and subscores difference before and after U-K test, and changes from baseline.

	Placebo		YRC3780	
	Baseline	Week 8	Baseline	Week 8
TMD score difference before and after U-K test	4.0 ± 4.4	2.9 ± 7.1	3.6 ± 3.4	–0.8 ± 5.5 *
Change in score from baseline (points)	—	–1.2 ± 6.4	—	–4.4 ± 6.2 *
Percentage change from baseline (%)	—	–22.8 ± 379.0	—	–167.6 ± 204.4*
AH score difference before and after U-K test	2.3 ± 5.3	0.3 ± 5.3	1.6 ± 5.2	–1.9 ± 5.1 *
Change in score from baseline (points)	—	–2.1 ± 5.6	—	–3.6 ± 6.9 *
Percentage change from baseline (%)	—	–28.5 ± 214.6	—	–78.2 ± 190.9
CB score difference before and after U-K test	3.5 ± 5.5	3.4 ± 7.7	3.8 ± 4.7	–0.3 ± 6.0 *
Change in score from baseline (points)	—	–0.1 ± 7.5	—	–4.1 ± 7.2 *
Percentage change from baseline (%)	—	2.0 ± 290.0	—	–64.6 ± 179.2
DD score difference before and after U-K test	3.1 ± 6.3	2.0 ± 6.6	1.9 ± 4.5	–2.0 ± 3.9 *
Change in score from baseline (points)	—	–1.0 ± 7.7	—	–3.9 ± 6.5 *
Percentage change from baseline (%)	—	38.1 ± 256.9	—	–117.5 ± 224.9 *
FI score difference before and after U-K test	6.0 ± 7.3	2.9 ± 7.8	5.6 ± 5.8	–0.2 ± 8.0 *
Change in score from baseline (points)	—	–3.1 ± 7.9	—	–5.9 ± 9.3 *
Percentage change from baseline (%)	—	–54.0 ± 129.4	—	–128.9 ± 282.0
TA score difference before and after U-K test	3.7 ± 6.0	2.0 ± 7.9	3.0 ± 5.8	–1.3 ± 7.2 *
Change in score from baseline (points)	—	–1.6 ± 7.3	—	–4.2 ± 7.1 *
Percentage change from baseline (%)	—	4.0 ± 387.8	—	–130.8 ± 259.4 *
VA score difference before and after U-K test	–1.7 ± 6.2	–3.4 ± 6.6	–2.0 ± 5.7	–2.7 ± 5.8
Change in score from baseline (points)	—	–1.7 ± 9.1	—	–0.7 ± 7.1
Percentage change from baseline (%)	—	–103.5 ± 347.1	—	–80.1 ± 327.1
F score difference before and after U-K test	–3.8 ± 7.5	–4.7 ± 6.3	–4.4 ± 7.6	–3.8 ± 5.4
Change in score from baseline (points)	—	–0.8 ± 8.8	—	0.6 ± 8.8
Percentage change from baseline (%)	—	–39.4 ± 186.1	—	–11.7 ± 153.0

Data are shown as means ± SD (Placebo, n = 53, YRC3780, n = 54).

Significant differences (*p < 0.05, ANCOVA with each initial value as a covariate) between the placebo and YRC3780 groups are indicated.

TA, Tension–Anxiety, DD, Depression–Dejection, AH, Anger–Hostility, CB, Confusion–Bewilderment, AH, Anger–Hostility, VA, Vigor–Activity FI, Fatigue–Inertia, F, Friendliness.

**Table 4:** POMS2 TMD (total mood disturbance) scores and subscores during intake period.

	Placebo			YRC3780		
	Beforethe U-K test			Before the U-K test		
	Baseline (n = 53)	Week 4 (n = 53)	Week 8 (n = 53)	Baseline (n = 55)*	Week 4 (n = 55)*	Week 8 (n = 54)
TMD score	56.9 ± 9.2	52.9 ± 9.3	50.3 ± 9.1	56.8 ± 11.2	52.1 ± 11.1	50.7 ± 10.7
Change in score from baseline (points)	—	–4.0 ± 9.2	–6.6 ± 7.8	—	–4.7 ± 8.8	–5.9 ± 9.0
Percentage change from baseline (%)	—	–5.9 ± 16.0	–10.8 ± 12.5	—	–7.5 ± 13.8	–9.5 ± 13.9
AH score	54.7 ± 10.0	52.7 ± 10.9	50.3 ± 11.4	53.2 ± 11.6	49.7 ± 9.6	48.9 ± 10.8
Change in score from baseline (points)	—	–2.0 ± 10.0	–4.4 ± 10.7	—	–3.5 ± 10.1	–4.3 ± 10.3
Percentage change from baseline (%)	—	–2.3 ± 18.4	–6.8 ± 19.7	—	–4.8 ± 16.0	–6.6 ± 16.2
CB score	57.7 ± 11.7	54.3 ± 11.5	50.4 ± 9.7	58.6 ± 12.0	54.2 ± 12.1	52.2 ± 11.2
Change in score from baseline (points)	—	–3.4 ± 11.8	–7.3 ± 8.1	—	–4.4 ± 9.6	–6.2 ± 9.8
Percentage change from baseline (%)	—	–3.9 ± 22.6	–11.4 ± 12.7	—	–6.5 ± 15.0	–9.3 ± 14.9
DD score	55.8 ± 10.4	52.3 ± 9.3	49.8 ± 8.1	55.8 ± 11.9	53.0 ± 10.6	50.8 ± 10.4
Change in score from baseline (points)	—	–3.5 ± 9.6	–6.0 ± 8.6	—	–2.8 ± 7.6	–5.0 ± 9.1
Percentage change from baseline (%)	—	–5.0 ± 15.1	–9.4 ± 13.4	—	–4.1 ± 11.7	–7.6 ± 13.3
FI score	56.1 ± 9.3	52.5 ± 9.9	50.8 ± 9.9	56.0 ± 11.1	51.5 ± 10.4	51.4 ± 10.5
Change in score from baseline (points)	—	–3.6 ± 7.9	–5.3 ± 8.3	—	–4.5 ± 9.0	–4.3 ± 9.4
Percentage change from baseline (%)	—	–5.6 ± 14.3	–8.6 ± 14.4	—	–6.7 ± 15.6	–6.4 ± 15.8
TA score	56.1 ± 9.4	50.7 ± 8.7	49.7 ± 8.3	57.0 ± 11.0	51.6 ± 10.9	51.1 ± 10.7
Change in score from baseline (points)	—	–5.4 ± 10.7	–6.4 ± 8.5	—	–5.4 ± 9.3	–5.7 ± 10.1
Percentage change from baseline (%)	—	–7.8 ± 18.9	–10.2 ± 15.0	—	–8.5 ± 15.3	–8.8 ± 15.8
VA score	47.0 ± 7.9	47.9 ± 10.8	49.1 ± 10.1	46.8 ± 9.1	49.7 ± 9.9	50.0 ± 9.9
Change in score from baseline (points)	—	1.0 ± 8.5	2.2 ± 7.6	—	2.9 ± 7.6	2.9 ± 6.4
Percentage change from baseline (%)	—	2.6 ± 18.4	5.1 ± 16.1	—	7.2 ± 16.1	7.0 ± 13.7
F score	50.7 ± 8.5	49.3 ± 11.2	49.9 ± 10.9	47.6 ± 11.3	47.9 ± 11.2	48.1 ± 8.8
Change in score from baseline (points)	—	–1.4 ± 8.1	–0.7 ± 8.1	—	0.3 ± 8.1	0.5 ± 6.2
Percentage change from baseline (%)	—	–2.5 ± 16.0	–1.1 ± 16.4	—	2.2 ± 18.7	3.3 ± 15.0

Data are shown as means ± SD (Placebo, n = 53, YRC3780, n = 54).

TA, Tension–Anxiety, DD, Depression–Dejection, AH, Anger–Hostility, CB, Confusion–Bewilderment, AH, Anger–Hostility, VA, Vigor–Activity FI, Fatigue–Inertia, F, Friendliness *Subjects who had been examined up to the 4 weeks were added to the baseline and 4 weeks analyses.

### Salivary cortisol

Salivary cortisol levels before, after, difference before and after, the U-K tests at baseline and 8 weeks, and its change from baseline, did not differ significantly between the YRC3780 and placebo groups (Table S4).

### VAS

VAS score for fatigue after, difference before and after the U-K tests at baseline and at 8 weeks, and its change from baseline, did not differ significantly between the YRC3780 and placebo groups (Table 5).

**Table 5:** VAS (visual analog scale) scores for fatigue after U-K test, and differences before and after U-K test.

	Placebo		YRC3780	
	Baseline	Week 8	Baseline	Week 8
VAS score after U-K test	67.3 ± 22.4	57.3 ± 21.1	66.6 ± 23.5	56.7 ± 23.6
Change in score from baseline (points)	-	–10.1 ± 23.7	-	–9.9 ± 23.3
VAS score difference before and after U-K test	15.0 ± 19.4	12.5 ± 15.2	12.2 ± 17.9	13.0 ± 15.6
Change in score from baseline (points)	-	–2.5 ± 21.1	-	0.8 ± 21.2

Change in score from baseline.

Data are shown as means ± SD(Placebo, n = 53, YRC3780, n = 54).

### DASS-21

The DASS-21 scores at baseline, 4 weeks and 8 weeks, and its change from baseline, did not differ significantly between the YRC3780 and placebo groups (Table S5). Subjects who had been examined up to the 4 weeks were added to the baseline and 4 weeks analyses.

### GHQ-28

The changes in the GHQ-28 scores at baseline, 4 weeks and 8 weeks, and its change from baseline, did not differ significantly between the YRC3780 and placebo groups (Table S6). Subjects who had been examined up to the 4 weeks were added to the baseline and 4 weeks analyses.

### BDI-2

The BDI-2 scores at baseline, 4 weeks and 8 weeks, and its change from baseline, did not differ significantly between the YRC3780 and placebo groups (Table S7). Subjects who had been examined up to the 4 weeks were added to the baseline and 4 weeks analyses.

## Discussion

The standardized (T) score used to evaluate POMS2 is a value that normalizes the metric; it has a mean value of 50 and a standard deviation of 10. The lower the T scores of TMD, TA, DD, AH, CB, and FI, which evaluate negative mood states, the more positive the mood state. In the case of VA and F, the higher the T score, the better the condition^14^. The primary outcomes, measured value of Total Mood Disturbance (TMD) in POMS2 after psychological stress load at 8 weeks, showed significantly lower values and changes in YRC3780 group compared to the placebo group (Table 1). In addition, regarding the other scores of POMS2 (TA, DD, AH, CB, and FI), YRC3780 group showed significantly lower values when compared to the placebo group (Table 2). Before and after the U-K test, the POMS subscores AH, CB, DD, FI, and TA decreased by an average of −0.2 to −1.9 (Table 3). The decrease in these scores may seems to that the U-K test contributes to mood states better. On the other hand, a study on the stress reduction effect of sniffing fragrances before the U-K test showed that even the control group aroma of water had a similar decrease of AH and DD by more than −1.0 before and after the U-K test. Furthermore, regarding CB, the control group increasing score, while the aroma group had a slightly decreasing score, indicating a significant difference. The above study anticipates an immediate relaxation effect from smelling fragrances, but such an effect is not expected in this research. Further studies are required to confirm reproducibility and to measure stress levels 30 min after the U-K test, comparing them to stress levels immediately afterward.

In previous study, it was confirmed that YRC3780 intake group, the salivary cortisol level 40 min after the TSST significantly decreased compared to the placebo group^8^. YRC3780 regulate the Th1/Th2 balance and induction of regulatory T cell^15^.In addition, a study by Slavich et al. in healthy adult men and women confirmed a significant increase in saliva IL-6 concentrations after the TSST^16^. It has also been reported that regulatory T cells decrease under acute stress loads such as the TSST and U-K test^17^. IL-6 is produced by Type 2 helper T cells (Th2 cells)^18^, but a previous study using a mouse model of atopic dermatitis confirmed that YRC3780 significantly reduced the concentration of IL-4 which is a marker of Th2 cells—in spleen cells, suggesting that it suppresses the activity of Th2 cells^19^. From these findings, it was inferred that YRC3780 reduced acute stress load through Treg induction and IL-6 suppression via the U-K test.

There were no significant differences between salivary cortisol,the VAS assessing fatigue,DASS-21 and BDI-2 which primarily assessed depressive symptoms. In previous studies, it was found that salivary cortisol levels increased due to TSST load, and in the YRC3780 group, the salivary cortisol levels at 40 min after the start of TSST significantly decreased compared to the placebo group. However, in this study salivary cortisol levels did not increase after the U-K test. There are reports indicating that the relationship between the U-K test load and cortisol in saliva did not change, similar to this study^20^, as well as reports that indicated an increase^21^, and no consistent conclusions have been reached. On the other hand, regarding reaction time to stress load, there are reports that amylase reacts quickly within 1 to several minutes^22^, whereas cortisol has a relatively long response latency of 20 to 30 min to stressors^21,23^. Shimizu et al. cortisol levels had already increased immediately after the U-K test compared to before the U-K test, but the peak occurred 20 to 30 min after the U-K test ended. Therefore, further research is needed. By increasing the number of saliva samples taken and measuring up to 30 min after the stress load, insights may be gained regarding the differences in reactivity between the TSST and U-K tests.

There are reports indicating that the fatigue VAS and POMS-2 FI show a good correlation^24^. However, Izawa et al. although there was no significant difference in FI in POMS-2, the mean values differed by more than 1.0, while the Average VAS scores were 39.2 and 39.7 without significant difference. Additionally, while VAS is a measure of fatigue, FI in POMS-2 differs in that it includes not only fatigue but also worn out, exhausted, weary and bushed. Therefore, while the tendency to shift negatively under stress is consistent, it is highly likely that the outcomes will not be the same.

In a study of American college men and women, individual differences in anxiety sensitivity were significantly correlated with the POMS2 TA score during stress loading, but no correlations were found with individual differences in anxiety traits^25^. In addition, the neural circuits that control anxiety and stress overlap, suggesting that there is a strong bidirectional relationship^26^. Our participants were selected to have relatively high TMD scores on POMS2 after U-K test at baseline, taking into account stress sensitivity, but not personality traits.

The Depression (D) subscale of POMS2 has been reported to be correlated with the total BDI-2 score^27,28^. Examination of the T scores for DD before stress load in our participants revealed that the mean score (estimated peripheral mean) and median score remained at about 50 points throughout the study period; as this value is considered to be average^14^, we inferred that the degree of depression was also average. In addition, over a time frame of more than 1 week, it is likely that the mood state specific to the situation at that time and the characteristics of that mood state will be mixed^14,29^. Whereas POMS2 has been shown to be useful over various time frames^14^, the DASS-21 questionnaire asks about a person’s state over the past week, so the influence of individual personality traits on the results of DASS-21 cannot be ruled out. Therefore, the lack of between- group significant differences in both DASS-21 and BDI-2, which were assessed only before the stress loading, may have been influenced by the personality traits of the trial participants.

Finally, our safety evaluations identified no adverse events in any of the participants throughout the study. Among those cases in which urinalysis and peripheral blood test measurements that were within the reference ranges at baseline fluctuated outside the reference ranges after the intervention, there were also some other items that were outside the reference values during the study period. However, for each group and item, the principal investigator confirmed that no medically problematic changes occurred upon consumption of the test meals (YRC3780 or placebo). Therefore, consumption of the test meals under the conditions of this study was safe.

The limitations of our study are that several cytokines including IL-6 were not measured, and that salivary cortisol, POMS2 and VAS for fatigue were only meseaed until immediately after U-K test. Further study is needed to explore the anti-stress and anti-fatigue effects of YRC3780, these parameters meseaed from 0 to 30 min after the U-K test.

This study found that daily intake of YRC3780 improves the stress response in healthy adults with a positive stress response in the U-K test.

## Methods

### Participants

Study participants were healthy Japanese adult men and women with increased POMS2 TMD scores after the U-K test at baseline and did not meet any of the exclusion criteria (Table 6). We enrolled 112 participants meeting these criteria and randomized two groups according to a computer-generated allocation table by the allocation officer. The allocation list was sealed in an envelope that was stored until the completion of data collection. The allocation was performed by allocation officer and concealed from the subjects, physicians, and researchers who recruited and assessed the participating subjects.

**Table 6:** Exclusion criteria.

1	History of, or under treatment for, malignancy, heart failure, or myocardial infarction
2	Implanted pacemaker or cardioverter defibrillator
3	Under treatment for arrhythmia, liver disorder, chronic kidney disease, cerebrovascular disease,rheumatic disease, diabetes, dyslipidemia, hypertension, or other chronic disease
4	Under treatment for chronic fatigue syndrome or menopausal disorder
5	History of depression, attention-deficit/hyperactivity disorder (ADHD), or other psychiatric disorder
6	Urinates 3 or more times during the night
7	Allergy to a pharmaceutical or food related to the test meal
8	Consumption of food for a specified health use or food with a functional claim
9	Use of pharmaceuticals (including herbal medicines) or dietary supplements
10	Alcohol consumption exceeding the daily average of approximately 20 g of pure alcohol (e.g., 500 mlbeer [one medium-sized bottle], 180 ml *sake*, 90 mL *shochu* [half a bottle], 60 ml whiskey or brandy (a“double”), 180 ml wine [about 1.5 glasses], 500 ml *chuhai* [about 1.5 cans])
11	Irregular sleep schedule or habits because of night shifts, etc
12	Irregular lifestyle habit (e.g., relating to diet, exercise, sleep)
13	Regular performance of heavy physical labor
14	Participation in another clinical trial during the 28 days before the date on which consent was obtainedfor the current study or is planned to occur during the current study
15	Deemed inappropriate for the study by the principal investigator

By using a document prepared by the study investigators, the study management staff of Orthomedico Inc. (Tokyo, Japan) explained the study to potential participants and, after confirming their full comprehension, obtained their voluntary informed consent to participate.

### Sample size

There has been no study to date that evaluates the impact of this test food on stress in healthy individuals based on the measured values of TMD after consumption for 8 weeks. Therefore, this study assumes that the difference in the measured values of TMD after the challenge between the test food group and the placebo group at the visit after 8 weeks of consumption is large, using Cohen’s suggestion of d = 0.80. The statistical significance level (α) was set at 0.05, and the statistical power (1 − β) was set at 0.90, calculating a required sample size of 68 participants (34 in each group). The target sample size was set at 100 participants (50 in each group). Additionally, to account for dropout and protocol violations during the trial period, the number of participants implemented was set to 112 (56 in each group).

### Test meal

The test meal was a capsule containing at least 5.9 × 10^10^ YRC3780 cells, cellulose, starch, and potassium stearate; placebo capsules did not contain YRC3780 but were otherwise the same. Both types of capsule were brown, thus preventing identification of the contents. The total cell count of YRC3780 in the test meal was confirmed by using a blood cell counter before the start of the study and through 4’,6-diamidino-2-phenylindole (DAPI) staining and quantitative PCR analysis at the end of the study.

### DNA extraction and PCR protocols

DNA extraction from the test meal was performed by using a Food DNA Isolation Kit (Norgen Biotek, Thorold, Canada) in accordance with the manufacturer’s instructions. Quantitative PCR analysis was performed by using the *L. cremoris* subsp. *cremoris –* specific primer LcCr-F, which was designed to target the 16S rRNA gene; the primer Lc-R, which detects most *Lactococcus* species^30^; and the LNA (locked nucleic acid) probe new-24base-LNA*3, to distinguish *L. cremoris* subsp. *cremoris* from the closely related species *L. cremoris* subsp. *tructae* (FASMAC, Kanagawa, Japan) (Table S1). Reaction mixture components and PCR condition were following at Table S2, S3.

### Study design

This randomized, double-blind, placebo-controlled, parallel-group trial was conducted from February through April 2024 in Japan. The 112 participants were assigned to either the YRC3780 group (*n* = 56) or the placebo group (*n* = 56). The participants were instructed to swallow one capsule (test meal or placebo) once daily for 8 weeks.

To evaluate subjective stress responses, before and after the U-K tests (baseline and 8 weeks) each participant completed the POMS2 test and had their salivary cortisol level measured. To evaluate fatigue, each participant completed a visual analog scale (VAS) before and after the U-K tests (baseline and 8 weeks). To evaluate normal mental status, each participant completed the BDI-II Beck Depression Questionnaire (BDI-2), the Depression, Anxiety and Stress Scale – 21 Items (DASS)−21, and the General Health Questionnaire – 28 Questions (GHQ-28) throughout the study (at baseline and at 4 and 8 weeks).

The study was conducted in accordance with the Declaration of Helsinki (revised version, 2013) and the Ethical Guidelines for Medical and Biological Research Involving Human Subjects as stipulated by the Ministry of Education, Culture, Sports, Science, and Technology; the Ministry of Health, Labor, and Welfare; and the Ministry of Economy, Trade, and Industry of Japan. It was approved by the institutional ethical review board at Seishinkai Medical Association Inc., Takara Clinic, on 18 October 2023. The study was registered in the University Medical Information Network Clinical Trial Registry (trial UMIN000052605. 25/10/2023).

### Efficacy outcomes and methods

The primary efficacy outcome was the POMS2 total mood disturbance (TMD) score after the U-K test at 8 weeks. The TMD score is calculated by adding the Tension-Anxiety (TA), Depression-Dejection (DD), Anger-Hostility (AH), Confusion-Bewilderment (CB), Anger-Hostility (AH), and Fatigue-Inertia (FI) subscores and then subtracting the Vigor-Activity (VA) subscore. The seven secondary outcomes were: a. the change in the POMS TMD score after the U-K test at 8 weeks from baseline and this percentage change; b. the POMS TMD score difference before and after the U-K test at 8 weeks, the change in score from the baseline, and percentage change from baseline; c. the POMS TMD score before the U-K test at 8 weeks, the change in score from the baseline, and percentage change from baseline; d. POMS2 (TA, DD, AH, CB, VA, FI, F) subscores, the VAS score for fatigue, salivary cortisol levels, after the U-K test at 8 weeks and difference before and after the U-K test at 8 weeks, the change in score from the baseline, and percentage change from baseline; e. POMS2 (TA, DD, AH, CB, VA, FI, F) subscores, the VAS score for fatigue, salivary cortisol levels, before the U-K test at 8 weeks, the change in score from the baseline, and percentage change from baseline; f. the DASS-21 scores for depression, anxiety, and stress, the GHQ-28 total score and the physical symptoms (A scale), anxiety and insomnia (B scale), social activity disorder (C scale), and depressive tendency (D scale) subscores, and the BDI-2 total score at 8 weeks, the change in score from the baseline, and percentage change from baseline; g. the POMS2 (TMD, TA, DD, AH, CB, VA, FI, and F), the VAS for fatigue score, the DASS-21 depression, anxiety and stress score, the GHQ-28 total score and physical symptoms (A scale), anxiety and insomnia (B scale), social activity disorder (C scale), and depressive tendency (D scale) subscores, and the BDI-2 total score at 4 weeks, the change in score from the baseline, and percentage change from baseline.

### Safety outcomes and methods

The primary safety outcome was the occurrence of adverse events. The secondary safety outcomes were the proportions of cases in which urinalysis and peripheral blood test results that were within the reference ranges at baseline exceeded them at 8 weeks. Other safety outcomes included physical measurements, physical examination data, and the results of urinalysis and peripheral blood tests.

### Statistical analysis

All statistical analyses were conducted by using IBM SPSS statistics software, version 23 and higher (IBM Japan, Tokyo, Japan). A *p* value of less than 0.05 was considered statistically significant. The study was designed to focus on the primary outcome and did not consider multiplicity occurring in the secondary outcomes, which were set up in a multihypothetical manner.For efficacy outcomes, differences between groups were evaluated by using Welch’s *t*-test and ANCOVA (analysis of covariance) with baseline values as covariates, as appropriate, with results reported as means ± standard deviation. The data set analyzed for efficacy outcomes was the full analysis set.

For the primary safety outcome, adverse events were tabulated by participant; incidence rates for adverse events were tabulated by group, and 95% confidence intervals were calculated for the incidence rates by group and for the difference in incidence rates between groups. The incidence of adverse events was compared between groups by using the chi-square test. For the secondary safety outcomes, we used the chi-square test to calculate the proportion of cases in which the urinalysis and peripheral blood test results that were within the reference values at baseline exceeded those limits afterward and to compare these data between groups by time point. For the other safety outcomes, summary statistics were calculated for each laboratory value. The data set analyzed for safety outcomes was the safety analysis population.

## Supplementary Information

Supplementary Information 1.

Supplementary Information 2.

Supplementary Information 3.

Supplementary Information 4.

Supplementary Information 5.

Supplementary Information 6.

Supplementary Information 7.

## Data Availability

The datasets generated during and/or analyzed during the current study are available from the corresponding author on reasonable request.

## References

[1] Reiwa 4 Occupational Safety and Health Survey https://www.mhlw.go.jp/toukei/list/dl/r04-46-50_gaikyo.pdf.

[2] Cryan, J. F. & Dinan, T. G. Mind-altering microorganisms: The impact of the gut microbiota on brain and behaviour. *Nat. Rev. Neurosci.* **13**(10), 701–712 (2012). 22968153 10.1038/nrn3346

[3] Kim, Y. K. & Maes, M. The role of the cytokine network in psychological stress. *Acta Neuropsychiatr.* **15**(3), 148–155 (2003). 26983358 10.1034/j.1601-5215.2003.00026.x

[4] Montiel-Castro, A. J., González-Cervantes, R. M., Bravo-Ruiseco, G. & Pacheco-López, G. The microbiota-gut-brain axis: Neurobehavioral correlates, health and sociality. *Front. Integr. Neurosci.* **7**, 70 (2013). 24109440 10.3389/fnint.2013.00070 PMC3791857

[5] Motoshima, H., Uchida, K., Watanabe, T., Tsukasaki F. Immunopotentiating effects of kefir and kefir isolates in mice. In *Proceedings of the 26th International Dairy Congress, the 26th IDF World Dairy Congress*. (Paris, Arilait Recherches, 2002).

[6] Uchida, K. et al. Effect of Lactococcus lactis subsp. cremoris YRC3780 on birch pollinosis: A randomized, double-blind, placebo-controlled clinical trial. *J. Funct. Food.* **43**, 173–179 (2018).

[7] Uchida, K. et al. Effect of drinkable yogurt containing Lactococcus lactis subsp. cremoris YRC3780 on symptoms of perennial allergic rhinitis ―a randomized, double‒blind, placebo‒controlled, parallel-group comparison study. *Jpn. Pharmacol. Ther.* **49**(7), 1165–1174 (2021).

[8] Matsuura, N., Motoshima, H., Uchida, K. & Yamanaka, Y. Effects of Lactococcus lactis subsp. cremoris YRC3780 daily intake on the HPA axis response to acute psychological stress in healthy. *Jpn. men. Eur. J. Clin. Nutr.* **76**(4), 574–580 (2022). 10.1038/s41430-021-00978-3 PMC8993685 34349248

[9] Godoy, L. D., Rossignoli, M. T., Delfino-Pereira, P., Garcia-Cairasco, N. & de Lima Umeoka, E. H. A Comprehensive overview on stress neurobiology: Basic concepts and clinical implications. *Front. Behav. Neurosis.* **12**, 127 (2018). 10.3389/fnbeh.2018.00127 PMC6043787 30034327

[10] Kirshbaum, C., Pirke, K. M. & Hellhammer, D. H. The ‘Trier Social Stress Test’–a tool for investigating psychobiological stress responses in a laboratory setting. *Neuropsychobiology* **28**, 76–81 (1993). 8255414 10.1159/000119004

[11] Kudielka, B. M., Schommer, N. C., Hellhammer, D. H. & Kirschbaum, C. Acute HPA axis responses, heart rate, and mood changes to psychosocial stress (TSST) in humans at different times of day. *Psychoneuroendocrinology* **29**, 983–992 (2004). 15219648 10.1016/j.psyneuen.2003.08.009

[12] Yamanaka, Y., Motoshima, H. & Uchida, K. Hypothalamic-pituitary-adrenal axis differentially responses to morning and evening psychological stress in healthy subjects. *Neuropsychopharmacol. Rep.* **39**(1), 41–47 (2019). 30480877 10.1002/npr2.12042 PMC7292277

[13] Maki, P. M. et al. Menstrual cycle effects on cortisol responsivity and emotional retrieval following a psychosocial stressor. *Horm. Behav.* **74**, 201–208 (2015). 26187711 10.1016/j.yhbeh.2015.06.023 PMC4876953

[14] Yokoyama, K. Japanese Manual of Profile of Mood States. In* 2*^*nd*^* eds. *(Kaneko Shobo, 2015).

[15] Nakagawa, R. et al. Lactococcus lactis subsp. cremoris YRC3780 modified function of mesenteric lymph node dendritic cells to modulate the balance of T cell differentiation inducing regulatory T cells. *Front. Immunol.* **8**(15), 1395380 (2024). 10.3389/fimmu.2024.1395380 PMC11261344 39040096

[16] Slavich, G. M. et al. Neural sensitivity to social rejection is associated with inflammatory responses to social stress. *PNAS* **107**(33), 14817–14822 (2010). 20679216 10.1073/pnas.1009164107 PMC2930449

[17] Freier, E. et al. Decrease of CD4 + FOXP3+ T regulatory cells in the peripheral blood of human subjects under going a mental stressor. *Psychoneuroendocrinology* **35**, 663–673 (2010). 20015595 10.1016/j.psyneuen.2009.10.005

[18] Kubo, M. Regulation of Th2 development and IgE antibody production. *Allergy* **62**(11), 1443–1450 (2013). 24552758

[19] Wang, D. et al. The effect of sleep duration and sleep quality on hypertension in middle-aged and older Chinese: The dongfeng-tongji cohort study. *Sleep Med.* **40**, 78–83 (2017). 29221783 10.1016/j.sleep.2017.09.024

[20] Izawa, N. et al. Stress reduction effect of aroma of whey fermented liquid. *J. Soc. Cosmer. Chem. Jpn.* **55**(2), 162–168 (2021).

[21] Shimizu, M., Higuchi, T., Kawakami, Y., Shimomura, H. & Shiba, K. Changes in salivary protein levels depending on the stress load. *J. Anal. Bio.-Sci.* **38**(3), 173–180 (2015).

[22] Takai, N. et al. Effect of psychological stress on the salivary cortisol and amylase levels in healthy young adults. *Arch. Oral Biol.* **49**(12), 963–968 (2004). 15485637 10.1016/j.archoralbio.2004.06.007

[23] Kudielka, B. M., Buske-Kirschbaum, A., Hellhammer, D. H. & Kirschbaum, C. HPA axis responses to laboratory psychosocial stress in healthy elderly adults, younger adults, and children: Impact of age and gender. *Psychoneuroendocrinology* **29**(1), 83–98 (2004). 14575731 10.1016/s0306-4530(02)00146-4

[24] Kobayashi, M., Hoshi, N. & Horiguchi, M. Validity of self-diagnosis fatigue checklist for young women. *Int. J. Hum. Cult. Stud.* **29**, 526–536 (2019).

[25] Shostak, B. B. & Peterson, R. A. Effects of anxiety sensitivity on emotional response to a stress task. *Behav. Res. Ther.* **28**(6), 513–521 (1990). 2076089 10.1016/0005-7967(90)90138-9

[26] Daviu, N. et al. Neurobiological links between stress and anxiety. *Neurobiol. Stress.* **11**, 1–9 (2019). 10.1016/j.ynstr.2019.100191 PMC6712367 31467945

[27] Griffith, N. M. et al. Measuring depressive symptoms among treatment-resistant seizure disorder patients: POMS Depression scale as an alternative to the BDI-II. *Epilepsy Behav.* **7**(2), 266–272 (2005). 16005686 10.1016/j.yebeh.2005.05.004

[28] Wang, Y.-P. & Gorenstein, C. Assessment of depression in medical patients: A systematic review of the utility of the Beck Depression Inventory-II. *Clinics* **68**(9), 1274–1287 (2013). 24141845 10.6061/clinics/2013(09)15 PMC3782729

[29] Rosenberg, E. L. Levels of analysis and the organization of affect. *Rev. Gen. Psychol.* **2**(3), 247–270 (1998).

[30] Odamaki, T. et al. Novel multiplex polymerase chain reaction primer set for identification of Lactococcus species. *Lett. Appl. Microbiol.* **52**, 491–496 (2011). 21299580 10.1111/j.1472-765X.2011.03028.x

