# Supplementary material for: Lactococcus cremoris YRC3780 improves subjective stress response in the Uchida-Kraepelin test: a randomized, double-blind, placebo-controlled study
Source: Sci Rep. 2025 Jul 2;15:23393. doi: 10.1038/s41598-025-07783-z (PMC12223139; doi:10.1038/s41598-025-07783-z)
Supplement: Supplementary file 1 — Supplementary Information 1. [file 41598_2025_7783_MOESM1_ESM.pdf]

Table S1. Primers and probe

Name of primer or probe	Sequence(5’-3’)
LcCr-F	TGCTTGCACCAATTTGAAGAG
Lc-R	GTTGAGCCACTGCCTTTTAC
new-24base-LNA*3	(6-FAM)_CCGCATAACAACTTTAAAC+A+T+AAG_(BHQ-1)

6-FAM, 6-carboxyfluorescein; BHQ-1, black hole quencher; +A and +T, locked nucleic acid (LNA)
