# Supplementary material for: Lactococcus cremoris YRC3780 improves subjective stress response in the Uchida-Kraepelin test: a randomized, double-blind, placebo-controlled study
Source: Sci Rep. 2025 Jul 2;15:23393. doi: 10.1038/s41598-025-07783-z (PMC12223139; doi:10.1038/s41598-025-07783-z)
Supplement: Supplementary file 2 — Supplementary Information 2. [file 41598_2025_7783_MOESM2_ESM.pdf]

Table S2. Components of reaction Mixture

	Volume
template DNA	2.5 μl
GenCheck qPCR Probe Master (dUTP) (FASMAC)	12.5 μl
primer LcCr-F (100 mM)	0.125 μl
primer Lc-R (100 mM)	0.125 μl
probe (10 mM)	0.5 μl
sterile distilled water	9.25 μl
Final Volume	25 μl
