# Supplementary material for: Lactococcus cremoris YRC3780 improves subjective stress response in the Uchida-Kraepelin test: a randomized, double-blind, placebo-controlled study
Source: Sci Rep. 2025 Jul 2;15:23393. doi: 10.1038/s41598-025-07783-z (PMC12223139; doi:10.1038/s41598-025-07783-z)
Supplement: Supplementary file 3 — Supplementary Information 3. [file 41598_2025_7783_MOESM3_ESM.pdf]

Table S3. PCR Condition

Step1	95 °C for 10 min
Step2	95 °C for 30 s→61℃ 1min / 40 cycles
