# Supplementary material for: Lactococcus cremoris YRC3780 improves subjective stress response in the Uchida-Kraepelin test: a randomized, double-blind, placebo-controlled study
Source: Sci Rep. 2025 Jul 2;15:23393. doi: 10.1038/s41598-025-07783-z (PMC12223139; doi:10.1038/s41598-025-07783-z)
Supplement: Supplementary file 4 — Supplementary Information 4. [file 41598_2025_7783_MOESM4_ESM.pdf]

Table S4. Salivary Cortisol before and after U-K test at baseline and 8weeks.

	Placebo		YRC3780	
	Baseline	Week 8	Baseline	Week 8
Salivary Cortisol before U-K test （μg/ dL）	0.2 ± 0.1	0.2 ± 0.2	0.2 ± 0.2	0.2 ± 0.1
Change in score from baseline	—	0.0 ± 0.2	—	0.0 ± 0.1
Percentage change from baseline (%)	—	16.3 ± 60.9	—	44.6 ± 114.4
Salivary Cortisol after U-K test （μg/ dL）	0.2 ± 0.1	0.2 ± 0.1	0.2 ± 0.1	0.2 ± 0.1
Change in score from baseline	—	0.0 ± 0.1	—	0.0 ± 0.1
Percentage change from baseline (%)	—	43.3 ± 83.6	—	27.8 ± 62.2
Salivary Cortisol difference before and after U-K test （μg/ dL）	0.0 ± 0.2	0.0 ± 0.2	-0.1 ± 0.1	0.0 ± 0.1
Change in score from baseline		0.0 ± 0.2		0.0 ± 0.1
Percentage change from baseline (%)	—	-132.4 ± 1164.0	—	189.2 ± 1102.6

Data are shown as means ± SD (Placebo, n=53, YRC3780, n=54).

Significant differences ($^{*}p < 0.05$, ANCOVA with each initial value as a covariate) between the placebo and YRC3780 groups are indicated.
