# Supplementary material for: Lactococcus cremoris YRC3780 improves subjective stress response in the Uchida-Kraepelin test: a randomized, double-blind, placebo-controlled study
Source: Sci Rep. 2025 Jul 2;15:23393. doi: 10.1038/s41598-025-07783-z (PMC12223139; doi:10.1038/s41598-025-07783-z)
Supplement: Supplementary file 5 — Supplementary Information 5. [file 41598_2025_7783_MOESM5_ESM.pdf]

Table S5. DASS-21 during intake period.

Scale	Placebo			YRC3780		
	Baseline (n=53)	Week 4 (n=53)	Week 8 (n=53)	Baseline (n=55)**	Week 4 (n=55)**	Week 8 (n=54)
Depression	6.5 ± 4.5	5.8 ± 4.3	4.5 ± 4.1	6.8 ± 5.1	5.3 ± 5.0	4.0 ± 4.4
Change in scale from baseline (points)*	—	-0.7 ± 3.6	-2.0 ± 3.7	—	-1.5 ± 3.7	-2.7 ± 4.2
Percentage change from baseline (%)*	—	0.0 ± 77.4	-27.7 ± 73.6	—	-14.1 ± 70.7	-38.2 ± 48.1
Anxiety	3.3 ± 3.3	3.0 ± 3.0	2.4 ± 2.8	3.9 ± 3.6	2.8 ± 3.3	2.3 ± 2.8
Change in scale from baseline (points)*	—	-0.3 ± 3.3	-1.0 ± 3.0	—	-1.1 ± 3.0	-1.6 ± 2.6
Percentage change from baseline (%)*	—	0.5 ± 92.7	-32.1 ± 77.1	—	-11.3 ± 73.2	-34.5 ± 62.0
Stress	6.5 ± 4.6	5.9 ± 3.9	4.9 ± 4.2	7.2 ± 5.5	5.4 ± 4.1	4.4 ± 4.2
Change in scale from baseline (points)*	—	-0.5 ± 3.9	-1.5 ± 4.1	—	-1.8 ± 3.6	-2.7 ± 3.8
Percentage change from baseline (%)*	—	10.0 ± 83.9	-18.0 ± 77.6	—	-12.8 ± 69.1	-32.1 ± 78.4

Data are shown as means ± SD (Placebo, n=53 YRC3780, n=54.)
*If the baseline value is 0, it is excluded from the analysis.
**Subjects who had been examined up to the 4 weeks were added to the baseline and 4 weeks analyses.
