# Supplementary material for: Lactococcus cremoris YRC3780 improves subjective stress response in the Uchida-Kraepelin test: a randomized, double-blind, placebo-controlled study
Source: Sci Rep. 2025 Jul 2;15:23393. doi: 10.1038/s41598-025-07783-z (PMC12223139; doi:10.1038/s41598-025-07783-z)
Supplement: Supplementary file 6 — Supplementary Information 6. [file 41598_2025_7783_MOESM6_ESM.pdf]

Table S6. GHQ28 during intake period.

	Placebo			YRC3780		
	Baseline (n=53)	Week 4 (n=53)	Week 8 (n=53)	Baseline (n=55)**	Week 4 (n=55)**	Week 8 (n=54)
Total score	10.5 ± 6.9	7.9 ± 6.3	6.7 ± 5.7	10.4 ± 6.9	8.2 ± 7.0	6.6 ± 6.3
Change in score from baseline	—	-2.5 ± 6.3	-3.8 ± 5.0	—	-2.2 ± 5.5	-3.5 ± 5.1
Percentage change from baseline (%)	—	-14.4 ± 68.9	-20.9 ± 67.4	—	-23.3 ± 46.6	-37.4 ± 41.4
Somatic Symptoms （A scale）	3.2 ± 2.2	2.7 ± 2.0	2.2 ± 1.8	2.9 ± 2.0	2.4 ± 2.1	2.4 ± 2.0
Change in score from baseline	—	-0.4 ± 2.0	-1.0 ± 1.9	—	-0.5 ± 1.9	-0.4 ± 1.7
Percentage change from baseline (%)	—	1.4 ± 89.8	-24.4 ± 73.3	—	-11.7 ± 75.1	-10.5 ± 71.0
Anxiety and Insomnia（B scale）	3.5 ± 2.2	2.5 ± 2.0	2.4 ± 2.0	3.7 ± 2.2	2.8 ± 2.1	2.5 ± 2.3
Change in score from baseline	—	-1.0 ± 2.2	-1.1 ± 2.0	—	-0.9 ± 2.0	-1.2 ± 2.2
Percentage change from baseline (%)	—	-21.3 ± 73.2	-20.6 ± 63.2	—	-28.1 ± 47.0	-33.6 ± 56.6
Social Dysfunction （C scale）	2.2 ± 2.1	1.4 ± 1.7	1.2 ± 1.7	2.3 ± 2.2	1.7 ± 2.1	1.1 ± 1.7
Change in score from baseline	—	-0.9 ± 2.2	-1.1 ± 1.9	—	-0.6 ± 2.1	-1.1 ± 1.9
Percentage change from baseline (%)	—	-11.7 ± 140.6	-45.9 ± 67.6	—	-19.2 ± 100.7	-48.2 ± 62.5
Severe Depression （D scale）	1.6 ± 2.1	1.3 ± 2.1	0.9 ± 1.8	1.5 ± 2.0	1.3 ± 1.9	0.7 ± 1.6
Change in score from baseline	—	-0.3 ± 2.1	-0.7 ± 1.4	—	-0.2 ± 1.3	-0.7 ± 1.2
Percentage change from baseline (%)	—	0.6 ± 166.7	-44.8 ± 85.9	—	-6.5 ± 76.8	-62.0 ± 49.2

Data are shown as means ± SD.
*If the baseline value is 0, it is excluded from the analysis.
**Subjects who had been examined up to the 4 weeks were added to the baseline and 4 weeks analyses.
