# Supplementary material for: Lactococcus cremoris YRC3780 improves subjective stress response in the Uchida-Kraepelin test: a randomized, double-blind, placebo-controlled study
Source: Sci Rep. 2025 Jul 2;15:23393. doi: 10.1038/s41598-025-07783-z (PMC12223139; doi:10.1038/s41598-025-07783-z)
Supplement: Supplementary file 7 — Supplementary Information 7. [file 41598_2025_7783_MOESM7_ESM.pdf]

Table S7. BDI-2 during intake period.

	Placebo			YRC3780		
	Baseline (n=53)	Week 4 (n=53)	Week 8 (n=53)	Baseline (n=55)**	Week 4 (n=55)**	Week 8 (n=54)
Total score	17.1 ± 10.3	12.2 ± 8.1	11.3 ± 9.1	17.7 ± 12.3	13.0 ± 10.2	11.2 ± 10.6
Change in score from baseline*	—	-4.9 ± 8.6	-5.8 ± 8.5	—	-4.7 ± 9.4	-6.3 ± 9.7
Percentage change from baseline (%)*	—	5.0 ± 142.6	-9.0 ± 118.1	—	-16.2 ± 63.0	-27.0 ± 61.2

Data are shown as means ± SD.
*If the baseline value is 0, it is excluded from the analysis.
**Subjects who had been examined up to the 4 weeks were added to the baseline and 4 weeks analyses.
